# Metabolomic Alterations in Thyrospheres and Adherent Parental Cells in Papillary Thyroid Carcinoma Cell Lines: A Pilot Study

**DOI:** 10.3390/ijms19102948

**Published:** 2018-09-27

**Authors:** Paola Caria, Laura Tronci, Tinuccia Dettori, Federica Murgia, Maria Laura Santoru, Julian L. Griffin, Roberta Vanni, Luigi Atzori

**Affiliations:** 1 Department of Biomedical Sciences, University of Cagliari, Cittadella Universitaria, 09042 Monserrato, Italy; paola.caria@unica.it (P.C.); lauratronci@unica.it (L.T.); dettorit@unica.it (T.D.); federica.murgia@unica.it (F.M.); marialaurasantoru@gmail.com (M.L.S.); latzori@unica.it (L.A.); 2 Department of Biochemistry and Cambridge Systems Biology Centre, University of Cambridge, Sanger Building, 80 Tennis Court Road, Cambridge CB21GA, UK; jlg40@cam.ac.uk

**Keywords:** cancer stem cells, papillary thyroid carcinoma, metabolomics

## Abstract

Papillary thyroid carcinoma (PTC), is characterized by a heterogeneous group of cells, including cancer stem cells (CSCs), crucially involved in tumor initiation, progression and recurrence. CSCs appear to have a distinct metabolic phenotype, compared to non-stem cancer cells. How they adapt their metabolism to the cancer process is still unclear, and no data are yet available for PTC. We recently isolated thyrospheres, containing cancer stem-like cells, from B-CPAP and TPC-1 cell lines derived from PTC of the *BRAF*-like expression profile class, and stem-like cells from Nthy-ori3-1 normal thyreocyte-derived cell line. In the present study, gas chromatography/mass spectrometry metabolomic profiles of cancer thyrospheres were compared to cancer parental adherent cells and to non cancer thyrospheres profiles. A statistically significant decrease of glycolytic pathway metabolites and variations in Krebs cycle metabolites was found in thyrospheres versus parental cells. Moreover, cancer stem-like cells showed statistically significant differences in Krebs cycle intermediates, amino acids, cholesterol, and fatty acids content, compared to non-cancer stem-like cells. For the first time, data are reported on the metabolic profile of PTC cancer stem-like cells and confirm that changes in metabolic pathways can be explored as new biomarkers and targets for therapy in this tumor.

## 1. Introduction

Thyroid carcinoma is the most common endocrine malignancy and papillary thyroid carcinoma (PTC) accounts for 85%–90% of all thyroid tumors [1,2,3]. Current knowledge in thyroid cancer relies on genetics, proteomics, molecular, and cell biology. Indeed, merging clinicopathological data with genomic alterations and transcriptome, proteome and methylome profiling, a molecular classification has recently been proposed [4]. Two major PTC subgroups were defined as “*BRAF*-like” and “*RAS*-like”, the first harboring driver mutations in *BRAF* gene, and *RET* rearrangements, with a very similar expression profile, the second characterized by *RAS*-mutation with different genomic, epigenomic, and proteomic features [5]. Moreover, telomerase reverse transcriptase (*TERT*) promoter mutations have been observed in 12–14% of PTC and associated with an extremely poor prognosis when accompanied by *BRAF^V600E^* mutation [6]. Nevertheless, there is still a need for exploring new tools to improve thyroid cancer detection rates, i.e., distinguishing between benign and malignant nodules, to avoid overtreatment and to accurately identify the nodules requiring more aggressive therapy. In this light, metabolomics is an emerging post-genomic holistic approach, addressing the systematic identification and quantitation of all metabolites in biological samples, including tumors. This new “omic” approach provides an insight of the entire set of metabolites, the so-called “metabolome”, in living systems, relying on different instrumental tools, such as mass spectrometry coupled with chromatographic techniques (MS-CG), nuclear magnetic resonance spectroscopy in conjunction with statistical techniques to define the discriminant metabolomic profile individually [7]. Metabolomic studies have demonstrated that biological pathways, including those involved in the production of energy, are highly modified in cancer, in comparison with normal differentiated cells, and have also contributed valuable information on thyroid carcinoma. For example, the potential role in discriminating different types of thyroid lesions, as well as in predicting lymph node (LN) metastasis in patients with papillary thyroid cancer (PTC) has been reported [8,9], These studies not only pinpointed the biological significance of metabolic alterations, but also indicated the potential role of metabolomic markers in developmental therapeutics. [10]. However, the presence of tumor heterogeneity remains an ongoing challenge. Indeed, as with most cancers, PTCs evolve by adapting to different micro-environmental conditions resulting in a tumor mass composed of genetically diversified cells. Within this heterogeneous population of cells, cancer stem cells (CSCs) are known to be the seed of tumor initiation, responsible for tumor occurrence, progression and therapeutic resistance [11], and consequently their characterization plays a key role in the understanding of cancer biology, especially in view of CSC-targeting therapies [12,13]. Mapping CSC metabolic phenotypes is a promising approach to targeting their metabolism. Although in vitro and in vivo studies have reported on the metabolic phenotypes of CSCs in a variety of tumors, such as breast [14], liver [15], pancreas [16] and ovarian cancer [17], the understanding of CSCs metabolism remains controversial and, to the best of our knowledge, no information is available on PTC.

We recently demonstrated that the B-CPAP [18] and TPC-1 [19] PTC-derived cell lines, representative of the “*BRAF*-like” subgroup [5], based on molecular driver categorization and histological diagnosis of the corresponding primary tumors, possess a subpopulation of cells with cancer stem-like properties capable of forming thyrospheres. We also demonstrated differences in functional properties, including the tumor-forming ability in nude mice [18], between thyrosphere cells, containing cancer stem-like cells and representing the early cancer-promoting subpopulation, and the corresponding parental adherent cells, representing the tumor bulk population. In the present pilot study we have used these PTC-derived cell lines to investigate putative differences in the metabolomic profile of thyrospheres and parental adherent cells, in order to shed some light in view of further ex vivo and in vivo studies. The B-CPAP cell line harbors *BRAF^V600E^*, *TP53,* and *TERT* promoter mutations and a certain degree of chromosome instability [18,20,21], and the TPC-1 has *RET/PTC1* and *TERT* promoter mutations [20,22]. We explored the metabolomic profiles using gas chromatography-mass spectrometry [GC-MS] and compared the results to define the discriminant metabolomic profile individually. Nthy-ory3-1 cell line, the only available cell line from thyrocytes negative for PTC-associated genetic mutations [23], used for functional studies [24,25] including metabolomics [26], was used as putative control. 

We found a substantial metabolic change between thyrospheres and adherent cells, showing an overlapping trend in both cancer cell lines. Our data indicate that metabolic alterations may contribute to the functional differences between these two tumor cell populations with different biological roles. Although the use of an in vitro model of PTC is a limit to our approach, the recent report that cancer cell lines distinctly mimic the metabolic gene expression pattern of the corresponding human tumors in liver [27] support the translation of our results, which for the first time indicate that cancer stem-like cells isolated from PTC-derived cell lines may be distinguished from the adherent cell population by a metabolomic approach, paving the road for in vivo studies. 

## 2. Results

### 2.1. Thyrospheres Forming Assay and Stemness Profile

**Figure 1:**
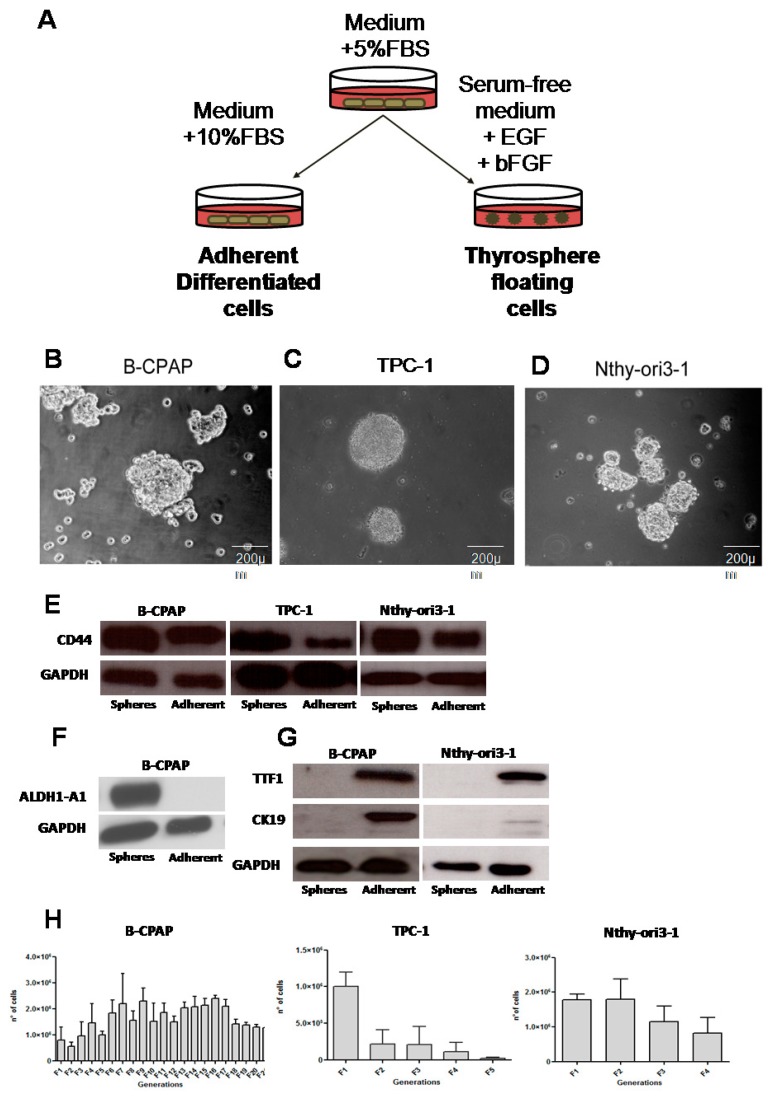
Thyrospheres forming assay. (**A**) The sphere-forming assay workflow. (**B**) A representative image of thyrospheres formed by B-CPAP cells at 7 days, 20×. (**C**) A representative image of thyrospheres formed by TPC-1 at seven days, 20×. (**D**) A representative image of thyrospheres formed by Nthy-ori3-1 at seven days, 20×. (**E**–**G**) Western blot analysis of stemness (ALDH1-A1, CD44) and differentiation markers (TTF1 and CK19) in adherent and thyrosphere cells. (**H**) Self-renewal assay of cell lines: Graphs show the total number of cells (Y axis) for each generations after 7 days of culture in serum-free medium.

Adherent cells were seeded in permissive conditions, at a density of 2 × 10^4^ cells/mL in serum-free medium (SFM) supplemented with epidermal growth factor (EGF) and basic fibroblastic growth factor (bFGF). Under these conditions, B-CPAP, TPC-1 and Nthy-ori3-1 cells were able to form thyrosphere in SFM. Cells began to form spheres on day 3 and reached their maximum after seven days of suspension culture (Figure 1A–D). 

Stemness markers were expressed in thyrospheres. CD44 was expressed in thyrospheres from both B-CPAP, TPC-1 and Nthy-ori3-1 cell lines (Figure 1E), while aldehyde dehydrogenase 1 (ALDH1) was expressed only in B-CPAP thyrospheres (Figure 1F). The TTF-1 transcription factor and the differentiation marker CK19 were detected only in B-CPAP adherent cells as well as in Nthy-ori3-1 cell but were negative in TPC-1 (Figure 1G). To evaluate the presence of self-renewing cells within thyrospheres, primary thyrospheres were enzymatically dissociated and re-seeded at the initial cell density. These cells were found to form secondary spheres. B-CPAP cells had an extensive capacity for self-renewal, reaching at least 21 generations (Figure 1H), whereas TPC-1 and Nthy-ori 3-1 self-renewal capacity was limited to five and four generations, respectively (Figure 1H).

### 2.2. Metabolomic Differences between Adherent Cells and Thyrospheres

The aqueous metabolites content of thyrospheres and adherent cells from B-CPAP, TPC-1 and Nthy-ori3-1 cells was characterized by GC-MS analysis. The growth medium was extracted in parallel to the cells for the measurement of extracellular lactate. Metabolites, including amino acids, sugars, organic and fatty acids were detected and reported as relative concentration of the metabolites, obtained by the chromatogram area and then normalized by total area. Afterwards, a supervised Partial Least Square-Discriminant Analysis (PLS-DA, allowing separation and distribution of the samples in the score plots based on the presence of discriminant metabolic variables) was performed to elucidate the discriminant metabolic features between thyrosphere and parental cells. Variable importance projection list (VIP), showing the most important variables over the models as a whole, was used to detect the metabolites responsible for the separation and these metabolites furthermore underwent a Student *t*-test.

#### 2.2.1. PTC-Derived B-CPAP

**Figure 2:**
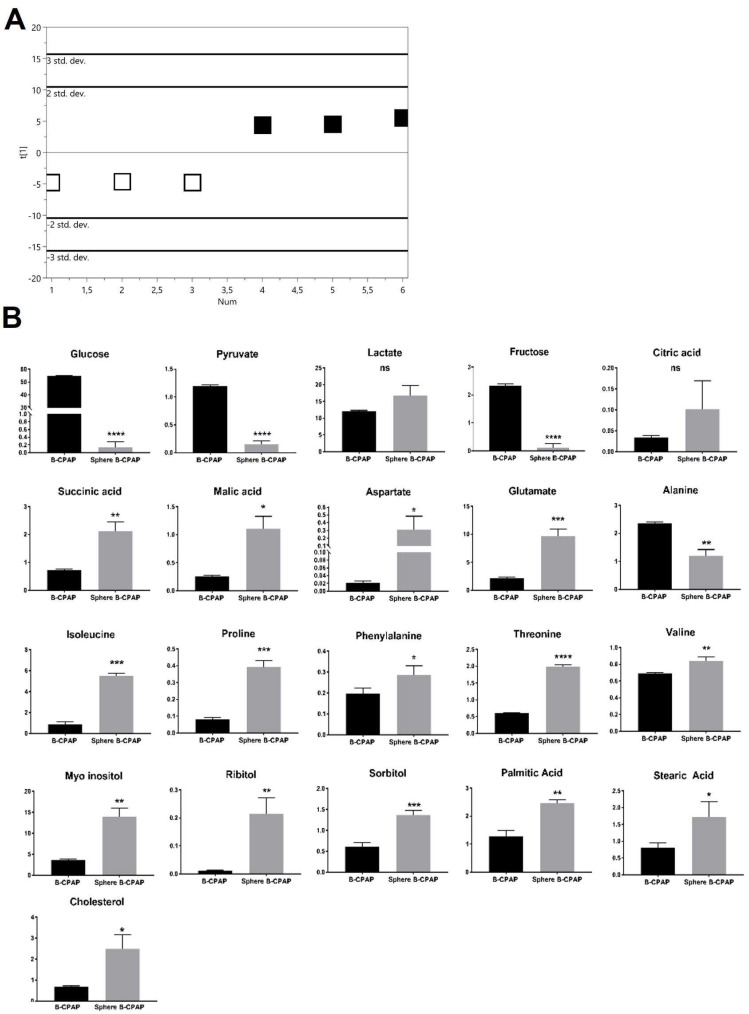
Metabolomic changes in B-CPAP derived thyrosphere and adherent cells. (**A**) PLS-DA scores plot resulting from metabolic profiles, showing distinct separation of the two cell populations (black squares: thyrosphere cells; white squares: adherent cells), based on the different metabolite concentrations. Model parameters: *R*^2^X = 0.495, *R*^2^Y = 0.942, *Q*^2^ = 0.854. (**B**) Bar graphs of the relative metabolite concentration. Statistical analyses were performed by unpaired Student *t*-Test. Data are presented as means ± standard deviation. All experiments were performed three times independently, each time in triplicate to confirm the results. * *p* < 0.05, ** *p* < 0.01, *** *p* < 0.001, **** *p* < 0.0001.

The score plot of the PLS-DA showed a clear separation between B-CPAP thyrospheres and adherent cells (Figure 2A) with good statistical parameters (*R*^2^X = 0.651, *R*^2^Y = 0.995, *Q*^2^ = 0.97) and the model was validated by permutation test (*n* = 200: intercepts: *R*^2^ = 0.01, *Q*^2^ = −0.474). Thyrospheres showed a significant decrease in glucose, pyruvate and fructose, all metabolites involved in glycolysis (Figure 2B). Among the discriminant metabolites that are direct or indirect components of the Krebs cycle, succinic acid, malic acid, aspartate, and glutamate were found significantly increased in B-CPAP thyrospheres (Figure 2B). Isoleucine, proline, phenylalanine, valine, and threonine were significantly increased in thyrospheres, while alanine was significantly decreased (Figure 2B). Myo-inositol and other polyols, such as ribitol and sorbitol, were significantly increased as well. Regarding lipids, palmitic acid, stearic acid, and cholesterol were also significantly increased (Figure 2B). 

#### 2.2.2. PTC-detived TPC-1

**Figure 3:**
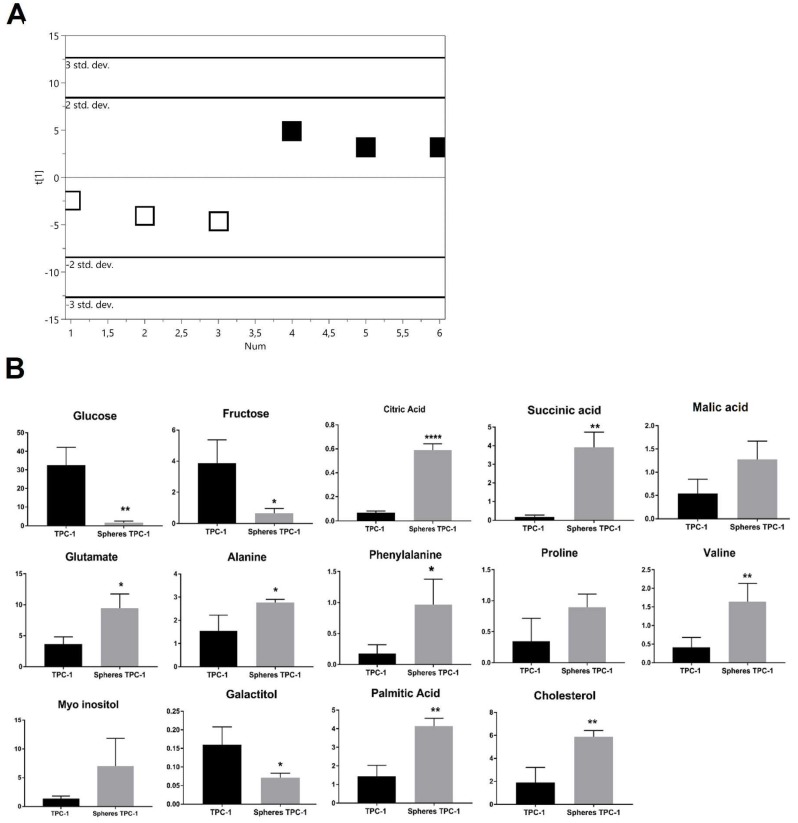
Metabolomic changes of TPC1 derived thyrosphere and adherent cells. (**A**) PLS-DA scores plot resulting from metabolic profiles, showing distinct separation of the two cell populations (black squares: thyrosphere cells; white squares: adherent cells), based on the different metabolite concentrations. Model parameters: *R*^2^X = 0.618, *R*^2^Y = 0.947, *Q*^2^ = 0.903. (**B**) Bar graphs of the relative metabolite concentration. Statistical analyses were performed by unpaired Student *t*-Test. Data are presented as means ± Standard Deviation. All experiments were performed three times independently, each time in triplicate to confirm the results. * *p* < 0.05, ** *p* < 0.01, **** *p* < 0.0001.

The score plot of the PLS-DA showed a clear separation between TPC-1 thyrospheres and adherent cells (Figure 3A) with good statistical parameters (*R*^2^X = 0.618, *R*^2^Y = 0.947, *Q*^2^ = 0.903) and the model was validated by permutation test (*n* = 200: intercepts: *R*^2^ = 0.294, *Q*^2^ = −0.385). Thyrospheres had a significantly decreased glucose and fructose, while citric acid, succinic acid and glutamate were significant increased. Alanine, phenylalanine and valine were also significantly increased in thyrospheres. Levels of galactitol were found to be significantly decreased, while palmitic acid and cholesterol were significantly increased again (Figure 3B).

#### 2.2.3. Thyrocyte-Derived Nthy-ori3-1

**Figure 4:**
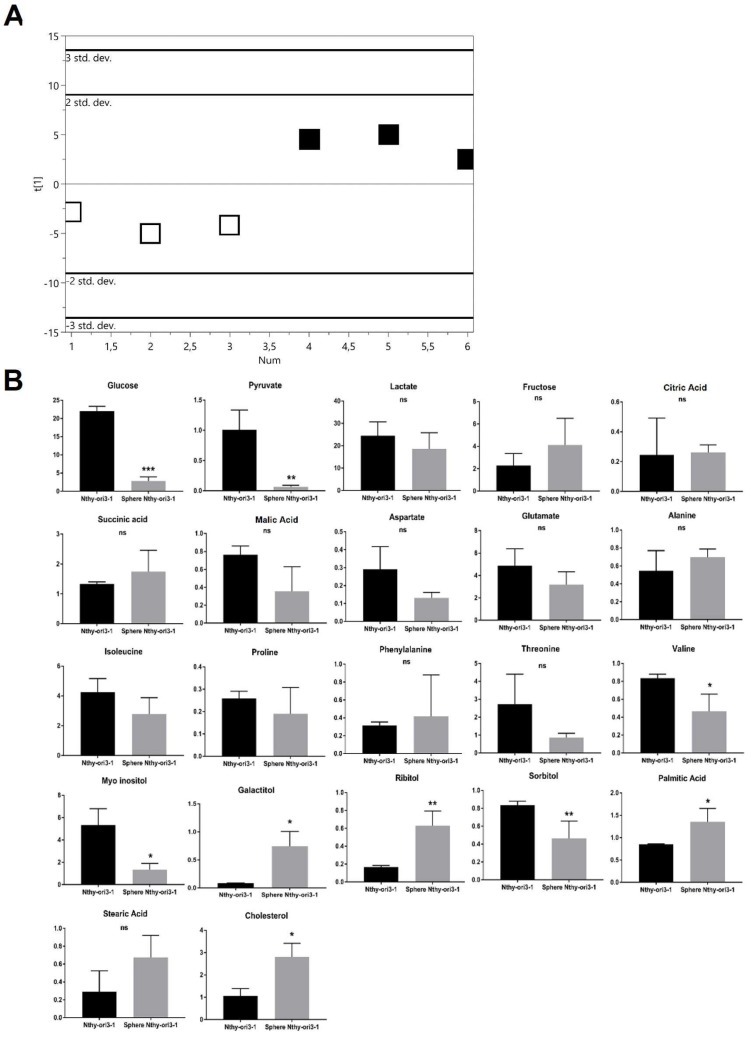
Metabolomic changes in Nthy-ori3-1 derived thyrosphere and adherent cells. (**A**) PLS-DA scores plot resulting from metabolic profiles, showing distinct separation of the two cell populations (black squares: Thyrosphere cells; white squares: Adherent cells), based on the different metabolite concentrations. Model parameters: *R*^2^X = 0.495, *R*^2^Y = 0.942, *Q*^2^ = 0.854. (**B**) Bar graphs of the relative metabolite concentration. Statistical analyses were performed by unpaired Student *t*-Test. Data are presented as means ± standard deviation. All experiments were performed three times independently, each time in triplicate to confirm the results. * *p* < 0.05, ** *p* < 0.01, *** *p* < 0.001.

The score plot of the PLS-DA showed a clear separation between Nthy-ori3-1 thyrospheres and adherent cells (Figure 4A) with good statistical parameters (*R*^2^X = 0.495, *R*^2^Y = 0.942, *Q*^2^ = 0.854) and the model was validated by permutation test (*n* = 200: intercepts: *R*^2^ = 0.0495, *Q*^2^ = −0.378). No significant difference was found for Krebs cycle metabolites, but valine, myo-inositol, cholesterol, ribitol, and galactitol were significantly increased, while palmitic acid and sorbitol were significantly decreased (Figure 4B).

### 2.3. Metabolomic Comparison of B-CPAP, TPC1 and Nthy-ori3-1 Thyrospheres

**Figure 5:**
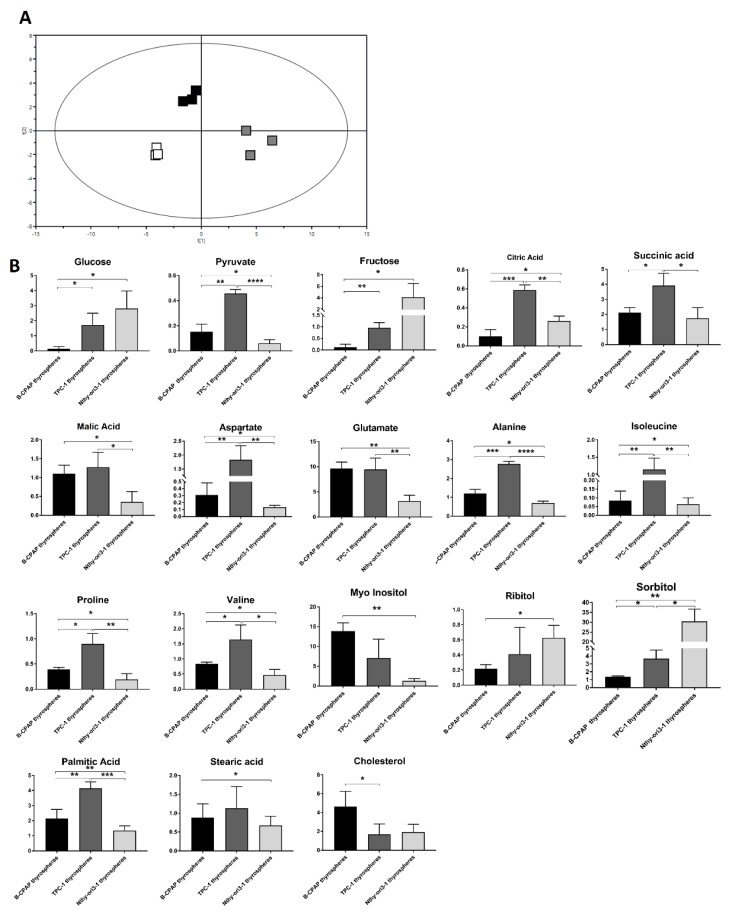
Metabolomic changes in B-CPAP, TPC-1 and Nthy-ori3-1 derived thyrosphere cells. (**A**) PLS-DA scores plot of metabolic profile of B-CPAP, TPC-1 and Nthy-ori3-1 thyrospheres. Model parameters: *R*^2^X = 0.738, *R*^2^Y = 0.949, *Q*^2^ = 0.998. (**B**) Bar graphs of metabolites, indicating the relative concentration of the metabolites obtained by the area under the peak in the chromatogram area and then normalized by total area. Statistical analyses were performed by Unpaired Student *t*-test. Data are presented as means ± standard deviation. All experiments were performed three times independently, each time in triplicate to confirm the results. * *p* < 0.05, ** *p* < 0.01, *** *p* < 0.001, **** *p* < 0.0001.

**Figure 6:**
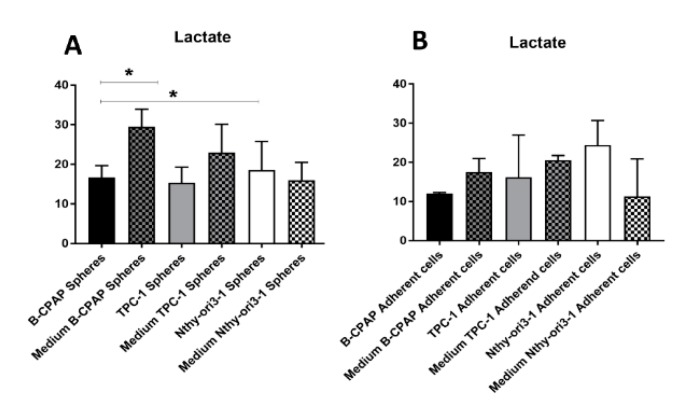
Lactate levels determination in growth medium of both adherent cells and thyrospheres. (**A**) Bar graphs of lactate levels in growth medium of thyrospheres from B-CPAP, TPC-1 and Nthy-ori3-1 (**B**) Bar graphs of lactate levels in growth medium of adherent cells from B-CPAP, TPC-1 and Nthy-ori3-1. Statistical analyses were performed by an unpaired Student *t*-test. Data are presented as means ± standard deviation. All experiments were performed three times independently, each time in triplicate to confirm the results. * *p* < 0.05.

**Figure 7:**
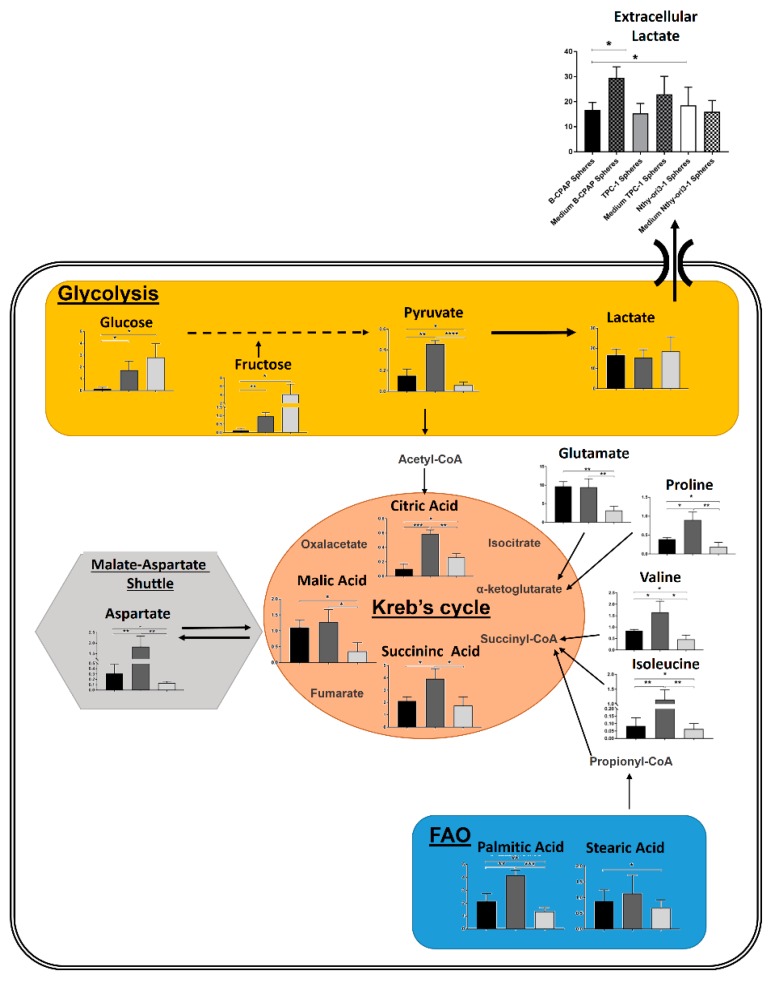
Summary of the most perturbed pathways involved in the metabolomics analysis of B-CPAP, TPC-1 and Nthy-ori3-1 thyrospheres (respectively black, grey and white bars). Variations in metabolites involved in glycolysis (glucose, fructose, pyruvate and lactate), linked to the lactate export in the extracellular space and to the metabolites involved in the Krebs cycle. Variation in citric, succinic and malic acids, involved in the Krebs cycle as well as associated changes in the amino acids, glutamate, proline, valine, isoleucine, which may fuel the cycle, all suggest a perturbed Krebs cycle flux. Palmitic and stearic acid levels involved in fatty acid oxidation (FAO) and aspartate levels, involved in the malate-aspartate shuttle further indicate metabolic deficits. * *p* < 0.05, ** *p* < 0.01, *** *p* < 0.001, **** *p* < 0.0001.

**Figure 8:**
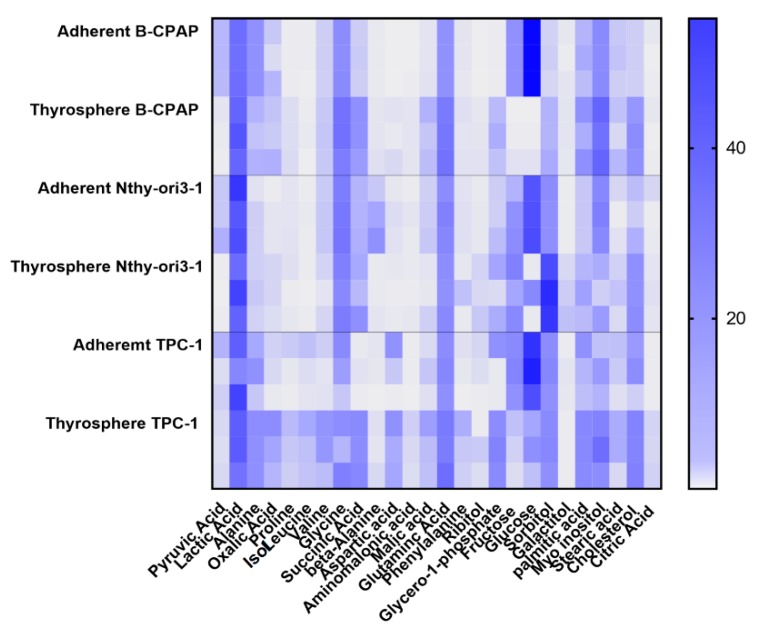
Metabolomic fingerprint of B-CPAP, TCP-1, NThy-ori3-1thyrospheres and adherent cells. Heat map of detected metabolites, in adherent and thyrospheres, reported as relative concentration.

A further PLS-DA model was performed among B-CPAP, TPC-1 and Nthy-ori3-1 derived thyrosphere cells. The score plot of the PLS-DA showed a clear separation among the three classes of thyrospheres (Figure 5A) with good statistical parameters (*R*^2^X = 0.738, *R*^2^Y = 0.949, *Q*^2^ = 0.998). Variations in metabolites levels, involved in glycolytic, TCA cycle, amino acids, polyols and fatty acids oxidation pathways are reported in Figure 5B. Furthermore, extracellular lactate was determined in the growth medium for both adherent cells and thyrospheres from B-CPAP, TPC-1 and Nthy-ori3-1 cell lines and shown in comparison with levels of intracellular lactate. Levels of extracellular lactate were significantly increased in the growth medium of B-CPAP thyrospheres compared to levels in B-CPAP thyrospheres (Figure 6). Based on the different metabolites detected from the analysis of B-CPAP, TPC-1 and Nthy-ori3-1 thyrospheres, a summary of the most perturbed pathways was built (Figure 7). The metabolic variation resulting from the instrumental analysis described above, is further summarized and highlighted in a heat map (Figure 8), showing the relative concentrations of all detected metabolites.

## 3. Discussion

Over the past two decades, increasing interest has focused on the cellular pool of CSCs, representing a subpopulation of tumor cells with self-renewal capacity, metastatic ability, therapeutic resistance and elevated metabolic plasticity [28]. The fundamental role of CSCs in tumor development and progression highlights the paramount importance of understanding of their metabolic dynamism, in view of developing combined strategies acting on CSCs metabolism, to antagonize their chemo-resistance, and metastatic ability [29]. Persistence of a small subpopulation of cancer stem-like cells is a feature of cancer cell lines [30]. Cancer stem-like cells enrichment can be achieved through tumorsphere cultivation, i.e., the formation of a sphere derived from a single cell in vitro, in the absence of serum and in conditions of low adhesion [31,32]. The assay has been widely accepted in studying CSC biology, including thyroid cancer biology [33]. In this pilot study, we investigated through a metabolomics approach the metabolic features of thyrospheres and adherent cells from the PTC-derived cell lines B-CPAP and TPC-1, harboring the molecular drivers of *BRAF*-like PTC sub-group, in comparison with a non tumoral thyreocyte-derived cell line. We observed a consistent difference in the metabolic profiles of thyrospheres versus parental adherent cells in all cell lines. Thyrosphere cells had a significant decrease of metabolites involved in the glycolytic pathway, e.g., glucose, pyruvate, and fructose, compared with parental adherent cells. This finding indicates that thyrospheres exploit a highly glycolytic metabolism (indicated by low concentration of glucose and fructose) and is in keeping with the observed high glycolytic phenotype of both CSCs and adult stem cells. Indeed, it has been shown that CSCs from breast [34], ovarian [35] and colon [36] cancers as well as normal stem cells [37] have a high energy demand which mostly depends on enhanced glycolysis. De facto, as expected, tumor thyrospheres, have a lower concentration of glucose and fructose than non tumor thyrospheres in keeping with the higher consumption of these metabolites, possibly leading to the higher proliferation rate and aggressive phenotype of cancer stem cells compared to non cancer stem cells. Moreover, our data also revealed that even though glycolysis was significantly increased in thyrospheres compared to adherent cells, metabolites from the Krebs cycle (such as succinic acid and malic acid) and metabolites which might fuel the Krebs cycle (such as aspartate and glutamate), were significantly increased only in cancer thyrospheres, indicating that cancer thyrospheres have an altered flux across the Krebs cycle, resulting in the accumulation of these metabolites. Taken together, these results support different metabolic energetics in cancer stem-like cells versus parental adherent cells as well as in tumoral versus non tumoral thyrospheres. The finding of a significantly increased level of aminoacids, detected only in cancer thyrospheres, suggests their role in fueling the Krebs cycle, determining an anaplerotic flux, a key aspect in proliferating cells [38]. Our data also show a significant increase in glutamate, in cancer thyrospheres. Intriguingly, glutamate has been previously reported to be significantly elevated following EMT induction [39], a phenomenon observed in TPC-1 and B-CPAP cells [40]. 

Some studies have reported that CSCs might also rely on mitochondrial processes, such as fatty acids oxidation [41,42]. The significant increase of fatty acids levels observed in thyrospheres, including palmitic and stearic acid, in B-CPAP and palmitic acid in TPC-1, suggests a fundamental role of fatty acid oxidation pathway in PTC biology, in keeping with the reported overexpression of genes associated with fatty acid oxidation pathways in CSCs isolated from ovarian cancer [43]. These data support the changes we observed in both B-CPAP and TPC-1 thyrospheres regarding the Krebs cycle. When cells increase fatty acid oxidation, intermediates in the second half of the cycle increase in concentration [44]. Furthermore, the observed amino acid changes in aspartate and glutamate concentrations observed in PTC-derived cells but not in thyrocytes, might be related to the alteration of the malate-aspartate shuttle. Indeed, the differential biological role of this shuttle in normal cells and cancer cells has been proposed and its function in preventing the glycolytic inhibition by high levels of cytosolic NADH/NAD+ ratios has been suggested [45]. Our finding also highlighted an increased level of succinic acid in the stem like cells from both B-CPAP and TPC-1, confirming its role as oncometabolite, in line with its critical role as mediator of the response to hypoxia [46], a potential contributor to the CSC phenotype [47]. The lactate excretion plays an important role in the tumor microenvironment, providing cancer cell metabolic fuel and activation of metastatic and angiogenic processes in cancer [48]. So, the increased levels of extracellular lactate in B-CPAP thyrospheres growth medium is of particular interest. 

In summary, our in vitro study showed alterations of several pathways in cancer thyrospheres compared to non-cancer thyrospheres. 

In conclusion, we report for the first time variations in the metabolism of cancer stem-like cells of two cell lines representative of the *BRAF*-like PTC subgroup in comparison to both non-stem cancer parental cells and thyrocytes stem-like cells.

The alterations particularly revealed changes in glycolysis, the Krebs cycle, and fatty acid oxidation metabolites, confirming the expected idea that cancer stem-like cells are characterized by metabolism distinct from the tumor bulk cells. However, while in other tumors the glycolytic and mitochondrial phenotypes are often mutually exclusive [12], in our model both glycolytic and oxidative metabolic pathways were altered, suggesting a possible metabolic plasticity. Although preliminary, our data, indicating identifiable metabolic features in thyroid cancer stem-like cells, contribute to the understanding of PTC cell biology and reinforces the idea that targeting cancer stem cells metabolism is a potential therapeutic tool.

## 4. Material and Methods

### 4.1. Cell and Sphere Culture

The human PTC-derived TPC-1and B-CPAP cell lines were kindly provided by Dr. Fusco (Medical School, University Federico II of Naples, Naples, Italy). Nthy-ori3–1Simian Virus 40 (SV40)-immortalized normal human thyrocytes were purchased from the Health Protection Agency Culture Collections. All cell lines were grown as a monolayer in culture in Dulbecco’s Modified Eagle’s Medium/Ham’s F-12 (DMEM/F12) supplemented with 10% fetal bovine serum (FBS, Life Technologies, Milan, Italy), 2 mM L-glutamine and 100 UI/mL penicillin and 100 μg/mL streptomycin (Sigma-Aldrich, Milan, Italy), at 37 °C in a humidified 5% CO_2_ atmosphere. A sphere-forming assay was used to generate thyrosphere cells (containing CSC-like cells) as previously described [18]. Briefly, adherent cells were gently enzymatically dissociated and single cells were cultured in permissive condition, at a density of 2 × 10^4^ cells/mL in low-attachment flasks (Corning, city, NY, USA), in serum-free medium (SFM) as follows: DMEM/F12 with 2% B27 supplement (Life Technologies) and epidermal growth factor (EGF), and basic fibroblastic growth factor (bFGF) (MiltenyiBiotec, Calderara di Reno, BO, Italy) (20 ng/mL each).To evaluate self-renewal, primary thyrospheres were enzymatically dissociated with StemPro Accutase (Life Technologies) and replated in SFM every seven days at a density of 2 × 10^4^ cells/mL to obtain next generation spheres. 

### 4.2. Immunoblotting

Cells were lysed with 2% sodium dodecyl sulfate at 4 °C. Protein concentration was measured according to the method of Lowry [49]. Western blot was carried out as previously described [18] using anti-CD44 (Thermo Scientific, Waltham, MA, USA) and anti-ALDH1A1 (Millipore, Temecula, CA, USA) anti-TTF1 (Abcam, Cambrige, UK) and anti-CK19 (Millipore) antibodies. Horseradish peroxidase-conjugated anti-mouse or anti-rabbit IgG (both from DAKO, Carpinteria, CA, USA) were used as secondary antibodies.

### 4.3. Aqueous Metabolites Extraction

Both thyrospheres and adherent cells were collected at a density of 4 × 10^6^ cells/mL. Prior to metabolite extraction, cells were washed with a physiological solution to ensure the removal of medium. Adherent cells were then scraped with a mixture of 1 mL of ice-cold methanol and water (80–20) and transferred in Eppendorf^TM^ tubes. Thyrosphere cells were centrifuged at 1300 rpm for 10 min and the cell pellet was reconstituted with 1 mL of cold methanol and water (80–20) for metabolite extraction. The extraction was combined with 10 min of ultrasonic treatment at 4 °C, to ensure the complete lysis of the cells. The lysate suspensions were then centrifuged at 4500 rpm for 30 min at 4 °C. The upper aqueous phase was separated, aliquoted in Eppendorf tubes and dried in a Concentrator Plus overnight (Eppendorf^TM^).

### 4.4. GC-MS Measurement of Metabolites

Dried pellets were derivatised with 50 μL of a solution of methoxyamine in pyridine (10 mg/mL) (Sigma-Aldrich, St. Louis, MO, USA). After 1 h at 70 °C, 50 μL of *N*-Methyl-*N*-(trimethylsilyl)-trifluoroacetamide, MSTFA, (Sigma-Aldrich, St. Louis, MO, USA) were added and left at room temperature for one hour. Successively, cells were re-suspended with 50 μL of hexane and transferred in vials for GC-MS analysis. Cell samples were injected splitless into a 7890A gas chromatograph coupled with a 5975C Network mass spectrometer (Agilent Technologies, Santa Clara, CA, USA) equipped with a 30 m × 0.25 mm ID, fused silica capillary column, which was chemically bonded with 0.25 μM TG-5MS stationary phase (Thermo Fisher Scientific, Waltham, MA, USA). The injector temperature was 250 °C. The gas flow rate through the column was 1 mL/min. Transfer line temperature was 280 °C. The column initial temperature was kept at 60 °C for 3 min, then increased from 60 °C to 140 °C at 7 °C/min, held at 140 °C for 4 min, increased from 140 °C to 300 °C at 5 °C/min and kept in isocratic mode at 300 °C for 1 min. Identification of metabolites was performed using the standard NIST 08 (http://www.nist.gov/srd/mslist.cfm), Fiehn 2013 (http://fiehnlab.ucdavis.edu/Metabolite-Library-2007) and GMD (http://gmd.mpimp-golm.mpg.de) mass spectra libraries and, when available, by comparison with authentic standards. Peak detection and deconvolution, filtering and normalization were performed using a pipeline in Knime [50]. Relative concentration of the discriminant metabolites was obtained by the chromatogram area and then normalized by total area.

### 4.5. Statistical Analysis

Multivariate statistical data analysis was performed using SIMCA version 14.0 (Umetrics, Umea, Sweden). To identify the presence of outliers, principal components analysis (PCA) coupled with Hotelling t-squared was used. Partial Least Square-Discriminant Analysis (PLS-DA) was performed to verify the metabolic differences between the thyrospheres and adherent cells. Variable importance in projection (VIP) lists was used to identify discriminant metabolites for the models and they were subjected to unpaired Student *t*-test with the GraphPad Prism v5.0 software (GraphPad Software, La Jolla, CA, USA). Data are presented as means ± standard deviation. All experiments were performed three times independently, each time in triplicate to confirm the results. Results were considered significant when * *p* < 0.05, ** *p* < 0.01, *** *p* < 0.001, **** *p* < 0.0001.
